# Effectiveness of secondary furlow palatoplasty with buccal myomucosal flap in correction of velopharyngeal insufficiency in patients with cleft palate

**DOI:** 10.1007/s00784-024-05607-4

**Published:** 2024-04-17

**Authors:** Mamdouh Ahmed Aboulhassan, Iman Mohamed Elrouby, Shaimaa Mohsen Refahee, Mohamed Abd-El-Ghafour

**Affiliations:** 1 Plastic Section, General Surgery Department, Faculty of Medicine, Cairo University, Cairo, Egypt; 2 Phoniatrics Department, Hearing and Speech Institute, Cairo, Egypt; 3 Oral and Maxillofacial Surgery Department, Faculty of Dentistry, Fayoum University, Fayoum, Egypt; 4 Department of Orthodontics, Faculty of Dentistry, Cairo University, Cairo, Egypt

**Keywords:** Two flap palatoplasty, Cleft palate, Velopharyngeal insufficiency, Furlow with buccal myomucosal flap palatoplasty

## Abstract

**Objectives:**

The main purpose of this study was evaluation of the effectiveness of secondary furlow palatoplasty with buccal myomucosal flap (FPBF) for the treatment of velopharyngeal insufficiency (VPI) in patients with a cleft palate who were treated with two flap palatoplasty (TFP) in their primary palate repair.

**Material and methods:**

Twenty-three medically free children aged 4–8 years with non-syndromic and previously repaired cleft palate via TFP participated in the study. All patients received secondary surgery following the technique of FPBF. Preoperative speech evaluation was done before the secondary repair and 3 months after the surgery using a hypernasal speech scale, speech intelligibility scale, and nasopharyngoscopy.

**Results:**

A statistically significant improvement was observed regarding the degree of hypernasality and speech intelligibility while comparing the preoperative scores after the primary surgery to the postoperative scores after the secondary surgery.

In addition, a statistically significant improvement was found in the nasopharyngoscopic assessment.

**Conclusions:**

The incorporation of a buccal myomucosal flap with Furlow palatoplasty was successful in improving hypernasality, speech intelligibility, and nasopharyngoscopic scores in patients with cleft palate.

**Trial registration:**

clinicaltrials.gov (NCT05626933).

**Clinical relevance:**

This technique might be the surgical technique of choice while treating patients who are suffering from VPI after cleft palate repair.

## Introduction

Cleft lip and palate is the most prevalent craniofacial birth defect [1]. After primary palatoplasty, there is a risk of development of multiple problems including velopharyngeal insufficiency (VPI). It was reported that VPI represents 5 to 35% of repaired cleft palate patients [2–6].

Velopharyngeal insufficiency in cleft patients is due to the abnormal closure between the pharyngeal wall and soft palate during speech which causes the nasal emission of the air during speech, nasal resonance, and abnormal articulation [7–10]. This abnormal closure is due to a short soft palate, improper positioning of the levator palatini, or surgical scar that limits the palatal muscles’ mobility [11, 12].

The most common techniques used for VPI management were pharyngeal flaps, or grafts as these improve the normal resonance by 61 to 76% [13, 14]. However, different studies reported that these techniques were followed by obstructive sleep apnea in 19–93% of the patients (13–15). Accordingly, palatoplasty techniques were defined as a preferred way for VPI management without endangering the upper airway [13, 14].

Although the two flap palatoplasty (TFP) was considered the most commonly used technique for primary palatoplasty. It is combined with VPI and speech problem as it offers inadequate soft palate length, divergent and non-functional levator palatini muscle [5, 16–19]. Several studies support that secondary Furlow palatoplasty may be the most preferred techniques for the management of VPI [20]. It enhances the palatal length and improves the levetor palatini muscle position and function [20, 21]. However, secondary Furlow palatoplasty cannot be used with moderate or severe VPI and velopharyngeal gap (VPG) > 5mm [22, 23]. To increase the efficacy of the Furlow technique in the severe cases, the incorporation of buccal myomucosal flap (FPBF) was suggested while dealing with VPI. It gives the soft palate more length than what produced that with Furlow palatoplasty alone.

The buccal myomucosal flap (BMMF) fills the palatal gap that normally filled with palate aponeurosis and decreases scar formation that pull the soft palate anteriorly. Aboulhassan et al. [24] reported that addition of buccal flap to Furlow Z-plasty improved the palatal length about 40% while Chang reported that Furlow Z-plasty alone increased the palatal length up to 30% only [24, 25].

Subsequently, the aim of the current study was to evaluate the effectiveness of the FPBF as a secondary surgery for the treatment of VPI in patients with cleft palate who were treated with 2-flap palatoplasty in their primary palate repair.

## Materials and methods

### Study design and setting

The current study is a cohort study carried out between November 2022 and August 2023. Fayoum University’s ethical committee granted ethical approval (EC2222). The study protocol that followed the Helsinki principles was submitted to clinicaltrialregistration.gov on November 25, 2022 (NCT05626933). All children’s guardians signed an informed consent after disclosing all processes and potential risks.

### Participants

Twenty-three medically free children aged 4–8 years with non-syndromic and previously repaired cleft palate via TFP participated in the study. All the primary cleft palate repair surgeries were completed at the age of 9–11 months by the same experienced surgeon. All the included children were with mild to moderate VPI as recorded before the secondary surgery.

All patients received a secondary surgery following the technique of FPBF. The same experienced surgeon completed all the secondary palatoplasties. An experienced speech language pathologist using perceptual speech assessment and nasopharyngoscopy did preoperative speech evaluation before the secondary repair and 3 months after the surgery.

### Sample size calculation

Sample size was calculated using data extracted from study done by Koh et al. [20]. From which the mean and standard deviation of the hypernasality scores were extracted and used in the calculation. The calculation was done using the G* power software with power set at the value of 80% while the level of significance was 0.05.

### Surgical procedures (Fig. 1a, b, c, d)

**Fig. 1:**
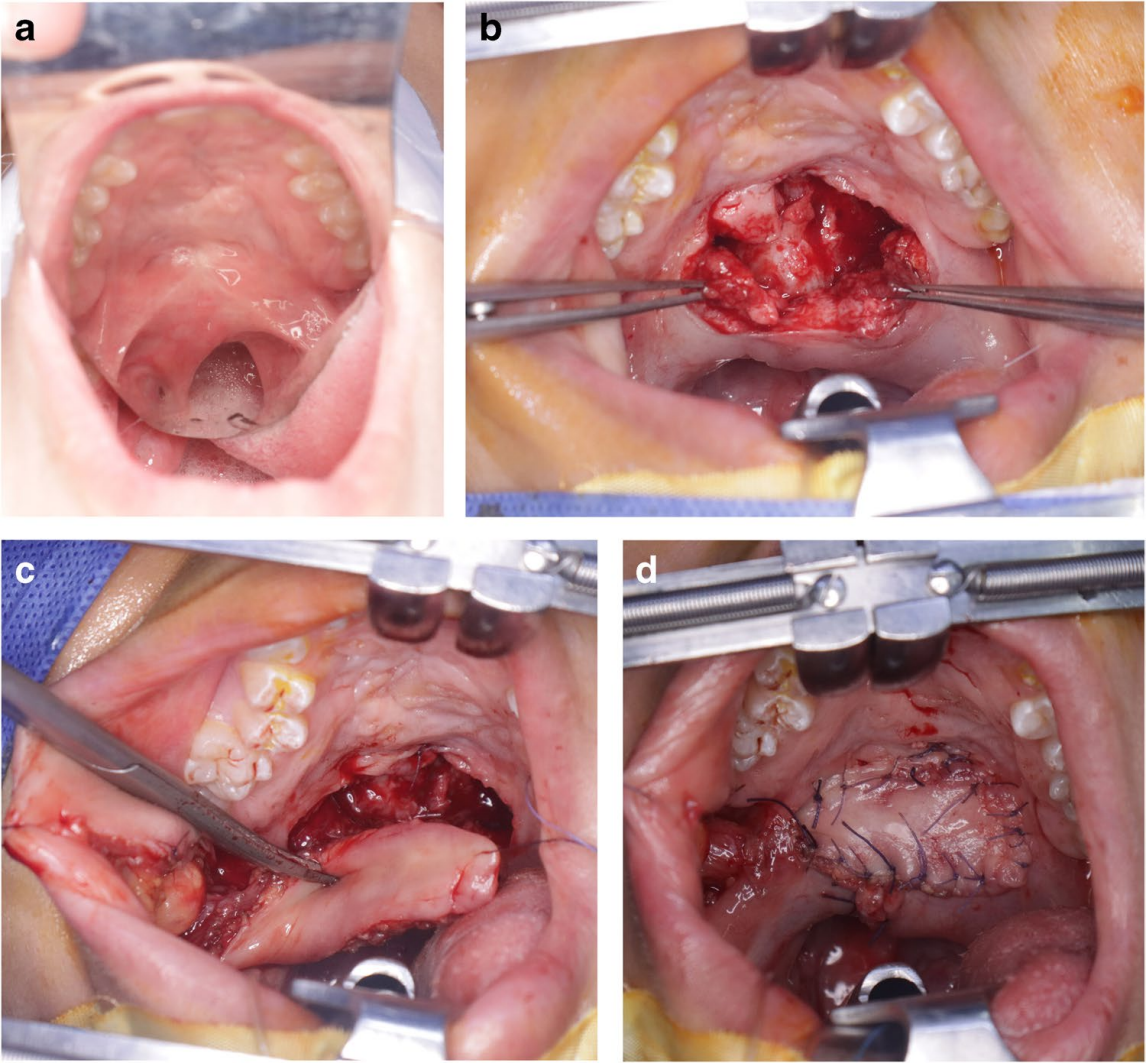
The surgical procedures of a secondary FPBF. **a**: pre-operative view of a short repaired palate. **b**: oral and nasal Z-plasty incision. **c**: showed the BMMF incision. **d**: postoperative view of the oral side residual defect closure by BMMF

After marking the Z-plasty design on the oral mucosa of the palate, local anaesthetic with vasoconstrictor was injected. The surgery started by incising the oral mucosa on the left side along the midline scar from the uvula till the junction of the hard with the soft palate including the levator palatini muscle. The incision was extended laterally toward the hamulus with an angle ranging between 60 -80 degrees to create a posteriorly based myomucosal flap. The levator muscle was completely detached from the hard palate and nasal mucosa. A small left anteriorly based nasal mucosal flap was done by cutting the nasal mucosa midway between the hard palate and uvula toward the head of the hamulus.

On the right side, an anteriorly based mucosal triangle was created by cutting the mucosa 5 mm above the base of the uvula toward the lateral edge of the hard palate. By blunt dissection, complete disinsertion of the muscle was done followed by cutting the nasal mucosa 8 mm distal the posterior bony edge of the hard palate to get a posteriorly based myomucosal flaps.

The nasal Z-plasty was interdigitated and sutured together. The oral side z-plasty then was closed with minimal underlying muscle overlapping. The residual defect was filled with buccal myomucosal flap.

A right BMMF was outlined on the right cheek inferior to the parotid duct with length 3.5 to 5.0 cm and width1.2 cm. The flap is raised by sharp dissection including the mucosa and majority of the muscle fibre leaving only a thin layer on top of the cheek fat. The flap was then rotated, tunnelled through the palatal mucosa, and sutured to the anterior edge of the z-plasty oral mucosa. Finally, the donor site was closed [24].

### Speech assessment

#### Perceptual speech assessment

Perceptual speech assessment was performed by recording the repeated different syllables, sentences and counting from 1 to 10 by the patients in a soundproof room. All the used sentences were in Arabic language. The records were assessed by the phoniatrician to detect the speech disorders as the severity of nasal resonance and speech intelligibility which were scored according to the hypernasality and speech intelligibility scale, respectively [26].

The hypernasality scale ranged from 0 to 3 (0 = no hypernasality, 1 = mild hypernasality, 2 = moderate hypernasality, 3 = severe hypernasality), similarly, the speech intelligibility scale ranged from 0 to 3 (0 = within normal limits, 1 = mild speech intelligibility, 2 = moderate speech intelligibility, 3 = severe speech intelligibility) [26].

#### Nasopharyngoscopic examination

After administering nasal topical anesthesia, all patients were examined using a flexible endoscope (Henke Sass Wolf- Gmbh Nasolaryngoscope, diameter 3.4 mm) that was coupled to a digital camera and was connected to a computer. The velopharyngeal valve was graded during speech (using syllables, sentences and counting from 1 to 10 in Arabic language) by a four-point scale according to the pattern of velopharyngeal closure, movement of the soft palate, lateral and posterior pharyngeal wall movement. In addition to the presence or absence of a VPG.

**Fig. 2:**
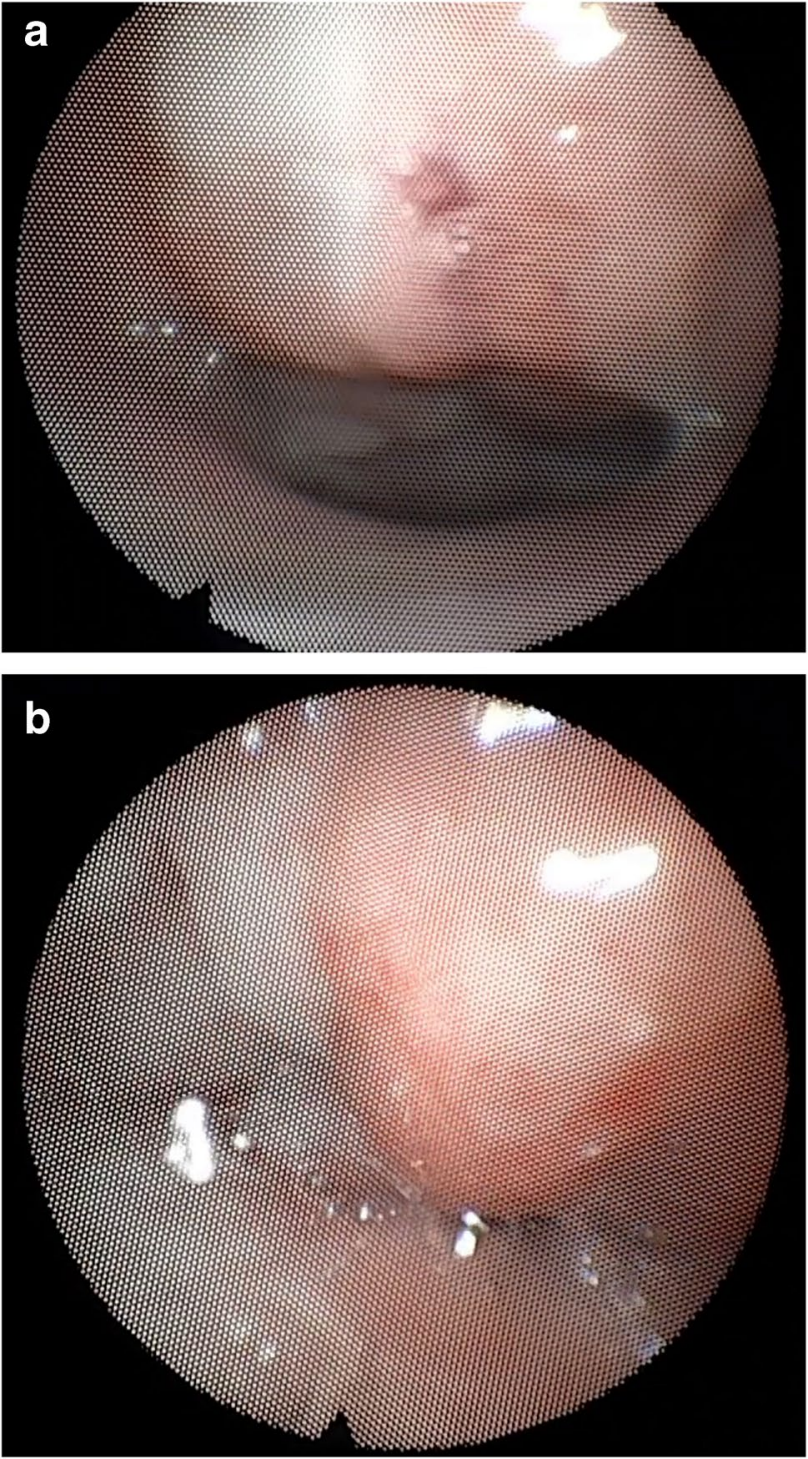
Showing pre- and post-surgical nasopharyngoscopic assessment during the speech. **a**: pre-surgical velopharyngeal port during speech (incomplete closure). **b**: post-surgical velopharyngeal port during speech (complete closure)

The four-point scale ranged from 0 to 4 with 0 = No closure, 1 = midway between 0–2, 2 = Incomplete closure (halfway closure), 3 = midway between 2–4, 4 = complete velopharyngeal closure [27] (Fig. 2a, b).

### Statistical Methods

The data obtained from the 3 assessment methods were tabulated using Microsoft Excel sheets. Mean and standard deviation were calculated for each assessment method at (T1) before the secondary surgery and at (T2) after the secondary surgery. Pre- and post-operative readings were compared using paired t-test while maintaining the level of significance at 0.05.

## Results

The current study included 23 children with non-syndromic repaired cleft palate aged 4–8 years that underwent secondary surgical palatal repair using FPBF. They were evaluated preoperatively and postoperatively after 3 months by perceptual speech assessment and nasopharyngoscopy (Table 1).

**Table 1:** Participant data

Cleft Type			Gender		Mean Age (years)
Cleft palate	UCLP		Male	Female	
	Right	Left			
9	5	9	8	15	6

UCLP; unilateral cleft lip and palate

The data of all the included 23 patients were analyzed. As shown in (Table 2), statistically significant improvements were observed regarding the degree of hypernasality and speech intelligibility while comparing the preoperative scores after the primary surgery (TFP) to the postoperative scores after the secondary surgery (FPBF).

**Table 2:** Comparison of hypernasality, speech intelligibilty pre- and post- operatively

Hypernasality							Speech Intelligibility						
Pre-operative (T1)		Post-operative (T2)		Difference		*P*-value	Pre-operative (T1)		Post-operative (T2)		Difference		*P*-value
Mean	SD	Mean	SD	Mean	SD		Mean	SD	Mean	SD	Mean	SD	
1.4	0.6	0.3	0.6	1.08	0.28	< 0.001*	1.3	0.5	0.3	0.4	1.09	0.27	< 0.001*

^*^ significant (*p* < 0.05)

A statistically significant improvement was found in the nasopharyngoscopic assessment as revealed in (Table 3) while comparing the preoperative scores to the postoperative scores 3 months later after FPBF. Significant improvement was observed in the grade of anteroposterior palatal and lateral pharyngeal wall mobility.

**Table 3:** Comparison of Nasopharyngoscopy scores pre- and post-operatively

Nasopharyngoscopy						
Pre-operative (T1)		Post-operative (T2)		Difference		*P*-value
Mean	SD	Mean	SD	Mean	SD	
2.8	0.4	3.9	0.3	1.04	0.20	< 0.001*

^*^significant (*p* < 0.05)

## Discussion

Two flap palatoplasty technique may be the favored technique for primary cleft palate repair [17]. It is an easy technique and can be used by the unexperienced surgeon [17]. In addition, it is associated with a low rate of fistula formation and an increase in the soft palate length by the push-pack theory [17]. In contrast, some patients managed with this technique suffered from VPI as Bardach et al. [5] and Cutting et al. [18] reported about 19% and 6% of repaired cleft palate patients suffered from VPI and speech problem. Dong F et al. [19] also reported that cleft palate repair by TFP was associated with speech problem and improper VP function. This may be attributed to the improper orientation of the levator muscle, inadequate soft palate length, non-perfect VP closure, and midline scar that impair the function of palatal muscle and VP function.

Velopharyngeal insufficiency is represented by nasality, unintelligibility of speech, abnormal articulation, and nasal emission of the air [8–10]. Abnormal articulation only can be managed by speech therapy but other manifestations need surgical correction of VPI [28, 29]. The most common surgical techniques used for VPI management were pharyngeal flaps, or grafts as these improve the normal resonance by 61 to 76% [13, 14]. However, different studies reported that these techniques were followed by upper airway obstruction in 19–93% of the patients [13–15]. Accordingly, palatoplasty techniques were used as choices for VPI management without endangering the upper airway [13, 14].

The incorporation of a BMMF with Furlow palatoplasty was used for the first time in 2017 as a primary palatoplasty to combine the advantages of both flaps [30]. Furlow with buccal myomucosal flap increases the soft palate length, and avoids the midline scar that contracts the soft palate away from the pharyngeal wall. In addition, it allows proper contraction of the levator muscle as it allows its proper repositioning and overlapping. All of these may contribute to the improvement of the VP function. Up to the moment, there is no study evaluating the effect of secondary FPBF on the management of VPI that occurs after primary cleft palate repair.

The null hypothesis of this study suggested that using a secondary FPBF is more effective in managing VPI post-cleft palate repair. Consequently, the aim of the study was to evaluate the use of secondary FPBF for the treatment of VPI post-cleft palate repair.

In the current study, the included patients were selected with age observed between 4–8 years old. The speech assessment began around 3 years ago as the normal child can cooperate during the speech assessment, and be able to repeat different sentences. In addition, the nasopharyngoscope cannot be used before 4 years. In most cleft centers, the VPI management was conducted around 4 years during the brain and speech development period and extended to 10 years if the VP function relapsed with growth [11, 31–33].

The selected patients in this study revealed significant improvement perceptually in hypernasality and speech intelligibility scores postoperatively. This is ascribed to good soft palate mobility and length that allow the proper contact between it and the pharyngeal wall. These results are in line with the study done by Mann et al.  [34] in which the patients had significant improvement in nasality and speech intelligibility scores that reached near normal levels postoperatively after primary cleft palate repair by FPBF.

Nasopharyngoscopy is a tool that is used nowadays in cleft centers to give information about the VP structure and function. In addition, it provides information about the size, shape, and location of the VP gap in the VPI cases [35]. Post-operative nasopharyngoscopic assessment in the current study showed a significant improvement in the grade of velopharyngeal closure as compared to preoperatively. This may be ascribed to the increase of the soft palate length and decrease the incidence of scar that affects the function of soft palate and pulls it away from the pharyngeal wall.

While considering the current study, evaluating the effectiveness of incorporating BMMF with Furlow palatoplasty as a secondary measure of VPI for the first time, the pre-calculated sample size, pre- and post-surgical evaluations are considered as points of strength. On the other hand, the short-term follow-up period followed might be considered as one of the limitations of the current study. Another limitation that stands in the current study is measuring the size of the original cleft defect, which was not considered. Including one group of patients can be considered as a strength and a limitation. As a strength, doing 2 different palatal repair techniques on the same group of patients excludes the inter-individual variability but of course, it will with higher risk of bias due to the followed study design. Some complications were recorded for using FPBF in primary cleft palate repair as buccal fat herniation, and buccal wound dehiscence [36]. However, in this study, no complication was observed throughout the follow-up period. Not comparing the BMMF with Furlow palatoplasty as a revision surgery to the use of Furlow alone is considered as one of the limitations of the current study and need further study to be assessed. Comparing Furlow with BMMF versus Furlow with buccal fat pad and Furlow alone in a future study will give a broader few on all the surgical options of the revision surgeries to treat VPI. Additionally, subgrouping of the included patients according to the need for revision surgery, cleft gap size and the type of the surgical technique should be considered in these future studies.

The incorporation of BMMF with Furlow palatoplasty can be considered a promising secondary intervention in patients with cleft palate to improve their speech capabilities.

Further studies will be needed to compare the effectiveness and rate of complications of secondary FPBF with other techniques used for VPI repair as pharyngeal flaps.

## Conclusions

The incorporation of BMMF with Furlow palatoplasty was successful in improving hypernasality, speech intelligibility, and nasopharyngoscopic scores in patients with cleft palate. In addition and within the limitations of the current study, this technique might be a surgical procedure with noticeable benefits while treating patients suffering from VPI after cleft palate repair.

## Data Availability

All datasets can be gotten from the corresponding author only.”

## Abbreviations

BMMF: Buccal Myomucosal Flap
FPBF: Furlow Palatoplasty with Buccal Myomucosal flap
TFP: Two Flap Palatoplasty
VPG: Velopharyngeal Gap
VPI: Velopharyngeal Insufficiency
